# Exploring the Nanostructures Accessible to an Organic Surfactant Atmospheric Aerosol Proxy

**DOI:** 10.1021/acs.jpca.2c04611

**Published:** 2022-09-28

**Authors:** Adam Milsom, Adam M. Squires, Isabel Quant, Nicholas J. Terrill, Steven Huband, Ben Woden, Edna R. Cabrera-Martinez, Christian Pfrang

**Affiliations:** † School of Geography, Earth and Environmental Sciences, University of Birmingham, Edgbaston B15 2TT, Birmingham, United Kingdom; ‡ Department of Chemistry, University of Bath, South Building, Soldier Down Ln, Claverton Down BA2 7AX, Bath, United Kingdom; § Diamond Light Source, Diamond House, Harwell Science and Innovation Campus, OX11 0DE, Didcot, United Kingdom; ∥ Department of Physics, University of Warwick, Coventry CV4 7AL, United Kingdom; ⊥ Department of Chemistry, University of Reading, Whiteknights, Reading RG6 6AD, United Kingdom; # Department of Meteorology, University of Reading, Whiteknights, Earley Gate, RG6 6BB, Reading, United Kingdom; ∇ School of Chemistry, University of Bristol, Bristol BS8 1TS, United Kingdom

## Abstract

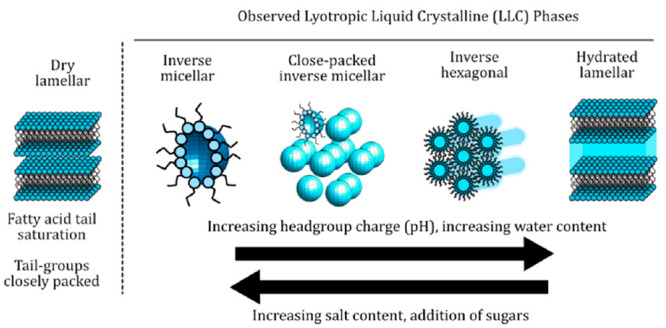

The composition of atmospheric aerosols varies with time, season, location, and environment. This affects key aerosol properties such as hygroscopicity and reactivity, influencing the aerosol’s impact on the climate and air quality. The organic fraction of atmospheric aerosol emissions often contains surfactant material, such as fatty acids. These molecules are known to form three-dimensional nanostructures in contact with water. Different nanostructures have marked differences in viscosity and diffusivity that are properties whose understanding is essential when considering an aerosol’s atmospheric impact. We have explored a range of nanostructures accessible to the organic surfactant oleic acid (commonly found in cooking emissions), simulating variation that is likely to happen in the atmosphere. This was achieved by changing the amount of water, aqueous phase salinity and by addition of other commonly coemitted compounds: sugars and stearic acid (the saturated analogue of oleic acid). The nanostructure was observed by both synchrotron and laboratory small/wide angle X-ray scattering (SAXS/WAXS) and found to be sensitive to the proxy composition. Additionally, the spacing between repeat units in these nanostructures was water content dependent (i.e., an increase from 41 to 54 Å in inverse hexagonal phase *d*-spacing when increasing the water content from 30 to 50 wt %), suggesting incorporation of water within the nanostructure. A significant decrease in mixture viscosity was also observed with increasing water content from ∼10^4^ to ∼10^2^ Pa s when increasing the water content from 30 to 60 wt %. Time-resolved SAXS experiments on levitated droplets of this proxy confirm the phase changes observed in bulk phase mixtures and demonstrate that coexistent nanostructures can form in droplets. Aerosol compositional and subsequent nanostructural changes could affect aerosol processes, leading to an impact on the climate and urban air pollution.

## Introduction

Aerosols are emitted into the atmosphere and affect the climate and human health.^1,2^ Their composition can vary with time, season, location, and environment.^3−7^ Aerosol components can be broadly split into an organic and inorganic fraction. An increased organic mass fraction has been linked to poor air quality.^8^ Additionally, some organic emissions are surface active and can decrease droplet surface tension and influence water uptake, affecting the ability of a particle to form a cloud droplet and therefore impacting on cloud formation and the climate.^9−12^

Oleic acid is a commonly emitted organic surfactant, with sources including marine^13,14^ and urban emissions.^15−17^ This has made it the compound of choice as a reactive organic surfactant aerosol proxy, e.g., see refs (18−26). Its surface-active nature can cause it to form self-assembled nanostructures when mixed with water and its salt (sodium oleate), these are called lyotropic liquid crystal (LLC) phases.^24,27,28^ These nanostructures can range from spherical micelles to lamellar multilayers and cylindrical arrangements with water channels, among others.^29^ Convention dictates that “inverse” LLC phases are where the surfactant tail is pointing away from the center of the structure, so-called “water-in-oil” phases. LLC phases bring with them a unique set of physical characteristics. Viscosity can vary by orders of magnitude when the surfactant nanostructure changes.^30^ Furthermore, diffusion becomes anisotropic for the inverse hexagonal (an array of cylindrical micelles) and lamellar phase, the latter with lateral diffusion coefficients orders of magnitude higher than in the orthogonal direction.^31^ This has implications for the diffusion through these nanostructures of small molecules such as water and atmospheric oxidants.

Aerosol viscosity and the diffusivity of small molecules within aerosol particles have a profound effect on their reactivity and ability to take up water.^32−37^ The phase state of atmospheric aerosols can range from liquid to semisolid and solid and is dependent on location, season, composition and meteorology.^38−41^ Phase state can also vary in indoor particles, suggesting aerosol viscosity and diffusivity could impact on indoor air quality.^42^ Knowing that atmospheric aerosol composition and phase state can vary in the atmosphere, there is potential for surfactant molecules (such as oleic acid) to form LLC nanostructures. The feasibility of LLC and organic crystalline phase formation in aerosol particles have been demonstrated in previous studies on a levitated oleic acid–sodium oleate proxy^20,24,43^ and the effect on reaction kinetics of the formation of one of these semisolid nanostructures (lamellar) was recently quantified; lamellar phase formation reduced reactivity by about an order of magnitude,^21^ potentially increasing the lifetime of oleic acid by days.^22^ These studies have contributed to explaining why oleic acid persists longer in the atmosphere compared with laboratory experiments, a long-standing issue in this field.^44,45^

This study seeks a better understanding of the nanostructures accessible to the oleic acid–sodium oleate–water organic surfactant aerosol proxy with the addition of other common atmospheric emissions (sugars and a saturated fatty acid) and by changing the amount and nature of the aqueous phase (i.e., changing the amount of water and the aqueous phase’s salinity). The oleic acid–sodium oleate system has been explored before in a biological context,^27,28,46^ and it has been studied qualitatively in an atmospheric context, demonstrating that nanostructure formation can occur in levitated droplets^24^ along with a crystalline organic phase.^20^ However, there is a lack of data concerning the systematic addition of atmospherically relevant molecules to the mixture. We achieve this by employing small angle X-ray scattering (SAXS), a powerful technique used previously to probe aggregate structures on the nanometer scale.^21,24,43^ A combination of laboratory and synchrotron SAXS experiments were carried out with the latter allowing for simultaneous wide-angle X-ray scattering (WAXS) and levitation of LLC phase mixtures. We observe and rationalize LLC phase changes and link them to variations in aerosol composition that can occur in the atmosphere, thus establishing potential atmospheric implications. This research also provides a resource for subsequent studies on the effect of surfactant (oleic acid) nanostructure on aerosol physical properties and in particular, but not exclusively, in an atmospheric context.

## Methods

The starting point for all self-assembled surfactant mixtures studied here are mixtures of oleic acid and sodium oleate, building on previous studies on this self-assembled atmospheric aerosol proxy systems.^20−22,24,43^ We define a “base” oleic acid–sodium oleate–aqueous phase bulk mixture where the organic fraction was made by mixing oleic acid (Sigma-Aldrich, 90% purity) and sodium oleate (Sigma-Aldrich, 99% purity) in a 1:1 wt ratio. Aqueous phase (deionized water or NaCl solution) was added to afford a final organic-aqueous ratio of 1:1 wt for this base mixture, which was used for all mixtures except when the oleic acid–sodium oleate weight ratio was specifically varied. Additional organic molecules (sugars and stearic acid) were added to the base mixture in the desired ratio (relative to the amount of oleic acid and sodium oleate) and the amount of aqueous phase was unchanged, except when this was the variable studied. (To clarify, an oleic acid–sodium oleate–fructose mixture at 1:1:1 organic wt ratio represents the original base mixture (oleic acid/sodium oleate/water {1:1:2 wt}) plus the additional fructose, representing 33 wt % of the organic fraction.) In this study, the wt % quoted when referring to a compound in a sample means the weight percentage of the organic fraction of the organic-aqueous mixture (e.g., 33 wt % fructose means fructose makes up 33 wt % of the organic fraction).

After adding the organic and aqueous components together, the samples were vortex mixed, heated to ∼50 °C for 20 min and vortex mixed again before being frozen in a conventional freezer. Samples appeared more homogeneous once frozen and thawed slowly at room temperature. Before the SAXS measurement, samples were allowed to reach room temperature and were once again vortex mixed before being sealed inside thin-walled glass X-ray capillary tubes of 1.5 mm internal diameter for the SAXS experiment.

**Figure 1:**
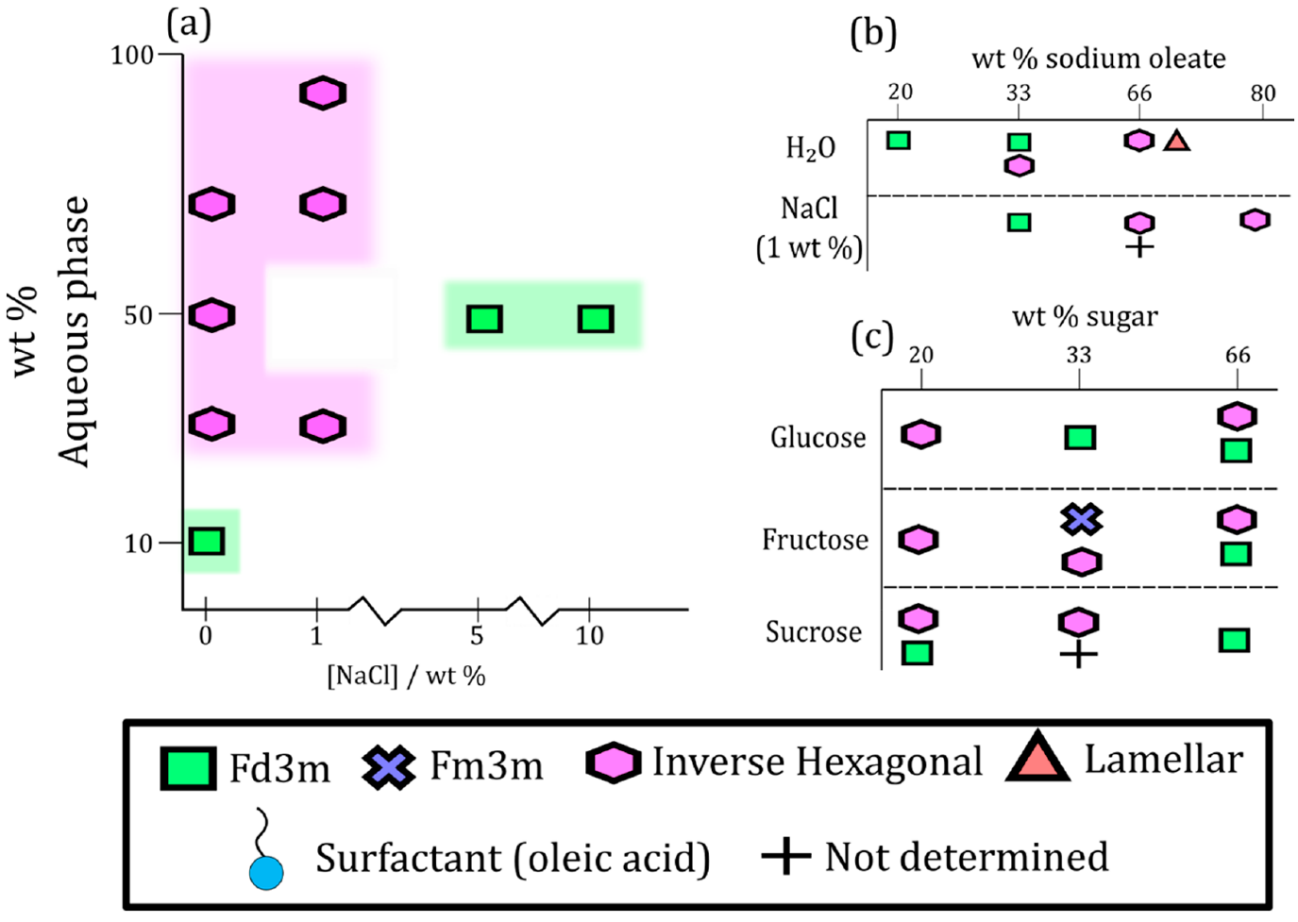
Phase diagrams derived from this study. (a) Changing the amount and salinity of the aqueous phase in the base mixture. (b) Changing the headgroup charge (wt % sodium oleate: SO) on the base mixture. (c) Changing the amount of sugar added to the base mixture.

SAXS is a technique used to probe aggregate materials on the nanoscale.^47^ Self-assembled LLC phases scatter X-rays to small angles and give scattering patterns with characteristic Bragg peaks.^48^ This scattered intensity is measured as a function of the scattering vector (**q**), which is inversely proportional to the spacing between equivalent scattering planes (*d*)
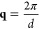
1This *d*-spacing can change with changing water content as water is gained or lost from the nanostructure.^49^ The relative position of each scattering peak for a given nanostructure is known and can be used to determine which nanostructure is present (see Figure 1 for example SAXS patterns for each observed phase and peaks labeled with their corresponding **q** position ratios).^48,50^

A combination of synchrotron and laboratory SAXS instrumentation was used to carry out the SAXS experiments at three different facilities as outlined below. Simultaneous WAXS was possible with the synchrotron setup, probing shorter repeat distances.

Synchrotron SAXS-WAXS experiments on bulk mixtures were carried out on the I22 beamline at the Diamond Light Source (U.K.). SAXS and WAXS patterns were measured by X-rays of 12.4 keV energy with 0.1 s collection times. The **q**-range for these SAXS and WAXS measurements were 0.008–0.46 and 0.4–0.6 Å^–1^, respectively. Samples were mounted in a sample rack and secured with Scotch tape.

Acoustic levitation-SAXS-WAXS experiments were also carried out on the I22 beamline. The levitation setup is described in detail in previous publications.^20,24^ Here, a microfocused X-ray beam of ∼14 μm (fwhm) diameter was used to acquire SAXS patterns from levitated droplets at 1 s acquisition time. The total SAXS-WAXS *q*-range for these experiments was 0.03–1.50 Å^–1^. Samples of oleic acid–sodium oleate (1:1 wt) in an excess NaCl (1 wt %) solution were injected into a pressure node of the acoustic levitator. Safety procedures at the beamline meant that SAXS experiments could not be started until ∼3 min after droplet injection.

An Anton Parr SAXSpoint 2.0 instrument was used to carry out the laboratory SAXS experiments. A Cu source produced X-rays of 1.54 Å wavelength. The sample-to-detector distance was 0.360 m and patterns were collected for ∼3–5 min for each sample. Samples were enclosed in Kapton capillaries of 1.5 mm internal diameter and mounted on a sample rack.

Further SAXS-WAXS experiments on mixtures with multiple additives were carried out on a Xenocs Xeuss 2.0 with a Cu X-ray source and a sample-to-detector distance of 1.185 m. A sample-to-detector distance of 0.162 m was used for WAXS measurements taken simultaneously with the SAXS measurements. Samples were mounted between two Kapton windows.

Rheological measurements were carried out using the HR-3 Discovery Hybrid Rheometer (TA Instruments) with a 40.0 mm 1.00694° cone plate geometry (Peltier plate Steel) and used to perform an oscillatory frequency sweep from 0.1 to 100 rad/s on the samples with a 0.2% strain applied at 25 °C. Dynamic viscosity of the samples was calculated by dividing the storage modulus by the radial frequency.

## Results and discussion

A qualitative summary of the phases observed in this study are presented in Figure 1a–c. By far, the most common phases observed are the inverse hexagonal and close-packing inverse micellar phases, often coexisting. All bulk mixtures studied here were probed by laboratory-based SAXS experiments except for the oleic acid-stearic acid mixtures, where synchrotron data were used.

The following discussion refers to interfacial curvature, more specifically negative interfacial curvature for the inverse phases studied here. This is the curvature at the water–surfactant headgroup interface.^48^ In this study, the phase with the largest negative interfacial curvature is the close-packed inverse micellar phase. The lamellar phase has zero interfacial curvature. In summary, the order from largest to smallest negative interfacial curvature is as follows: inverse micellar; close-packed inverse micellar; and inverse heagonal and lamellar.

**Figure 2:**
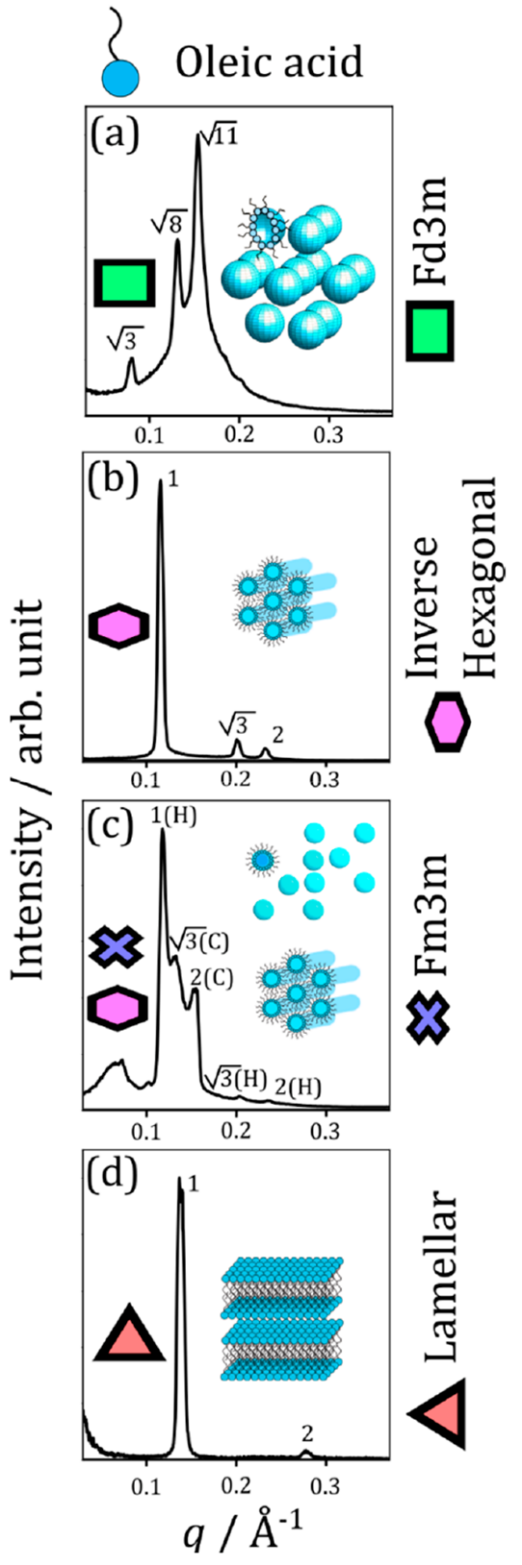
Typical 1D SAXS patterns for the fatty acid nanostructures observed in this study. (a) Cubic close-packed inverse micelles (*Fd*3*m* symmetry). (b) Inverse hexagonal phase. (c) Mixture of cubic close-packed inverse micelles (*Fm*3*m* symmetry) and inverse hexagonal phase. (d) Lamellar bilayers. Cartoons of each of these phases are also presented (not to scale). Each peak is labeled with the peak position ratio expected for each phase, i.e., for the lamellar phase in panel (d), the second peak appears at 2× first peak position value (in **q**). For panel (c), inverse hexagonal and cubic phase peaks are labeled (H) and (C), respectively.

After collection of the 2D SAXS pattern from each sample, a radial integration was performed over the *q*-range to afford a 1D scattering pattern. The 2D SAXS patterns did not exhibit any LLC phase orientation. Examples of scattering patterns for the phases observed in this study are presented in Figure 2. Cartoons of each phase are also presented. Note that coexisting phases were determined by matching peak positions to those predicted for the phases observed here (Figure 2a–d).

### Changing the Nature of the Surfactant

**Figure 3:**
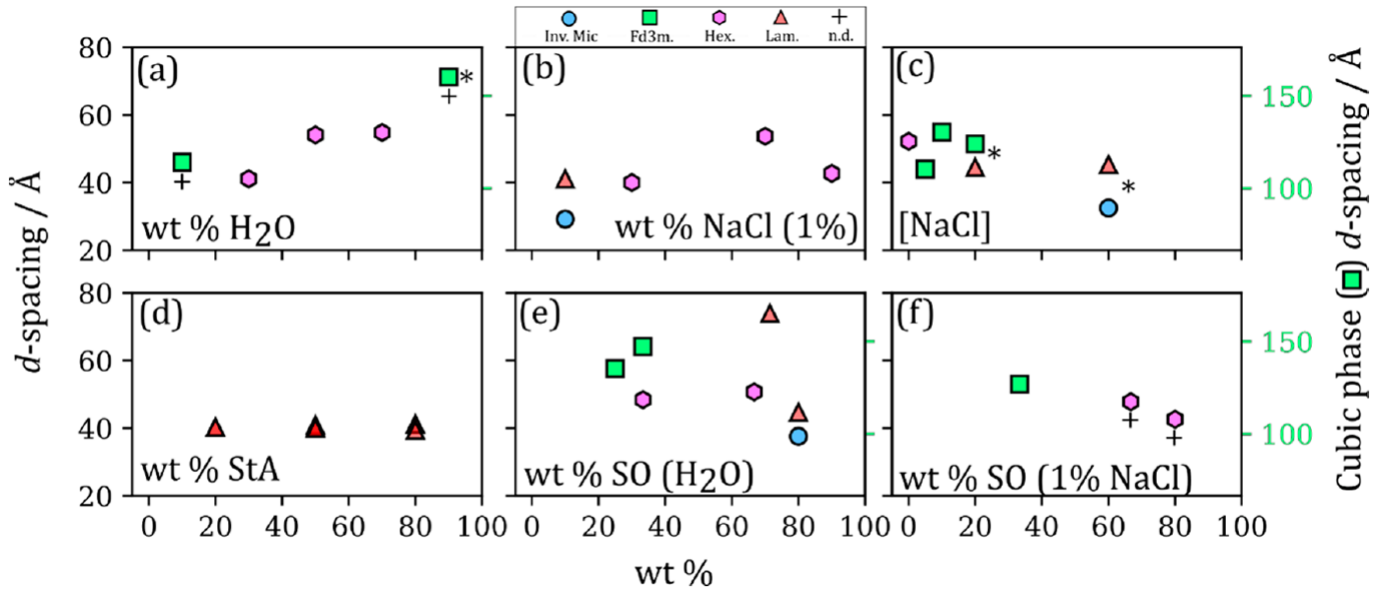
Plots of observed nanostructures and their calculated *d*-spacings vs wt % of each component added to the base mixture: oleic acid/sodium oleate (1:1) 50% wt in H_2_O except for the experiment series varying wt % H_2_O, wt % NaCl (1%) solution and wt % StA. (a) wt % H_2_O; (b) wt % NaCl (1%); (c) [NaCl]; (d) wt % StA; (e) wt % SO in H_2_O; (f) wt % SO in NaCl (1%) solution. The *d*-spacings for the cubic (*Fd*3*m* symmetry) nanostructure are placed on a different scale (right-hand side) due to the large difference in values compared to the other nanostructures. StA, stearic acid (saturated oleic acid analogue); SO, sodium oleate. Phases are abbreviated: Inverse micellar (Inv. Mic.); *Fd*3*m*-symmetry cubic close-packed inverse micelles (*Fd*3*m*); inverse hexagonal (Hex.); lamellar (Lam.); not determined due to overlapping peaks (n.d.). An “*” represents mixtures where phase separation was observed visually.

Oleic acid is typically found in organic emissions together with its saturated analogue, stearic acid.^17^ The relative proportions of these molecules can vary and can be a measure of how much a sample has aged.^4^Figure 3d shows that the equilibrium phase observed for dry mixtures of oleic acid and stearic acid is the lamellar phase. One can assume that this is a crystalline structure due to the lack of water in the system and this is confirmed by the peaks we observed in the wide-angle X-ray scattering (WAXS) region (see Figure S1, SI). Note that the *d*-spacing for all oleic acid-stearic acid compositions is slightly smaller (by 3–4 Å) than that of the dry oleic acid–sodium oleate mixture (Table S1, SI). This suggests that the alkyl chain monolayers of the lamellar bilayer can pack closer together, which is likely due to the lack of a kink in the stearic acid alkyl chain compared to the one induced by the cis double bond in oleic acid. The presence of WAXS peaks supports this since these peaks arise from well-packed adjacent alkyl chains.^51^

The availability of the double bond to the atmospheric oxidant ozone is likely to be affected by the spacing between alkyl chains by reducing the diffusivity of ozone through the organic phase, limiting the reaction to the surface layers (at least initially),^21^ a hypothesis also described by Hearn et al.^52^ This may also explain the trend in reactivity observed by Katrib et al., who reacted oleic acid-stearic acid particles with ozone. They observed that the ozonolysis reaction effectively stopped at high stearic acid compositions.^53^ Though explanations were offered, no experiments on the nanostructure of those particles were carried out; our work provides experimental evidence suggesting that the formation of these tightly packed lamellae could play a role in determining reactivity. The difference in fatty acid “steric configuration” has also been suggested to explain the difference in reactivity between the trans- and cis*-* isomers of oleic acid observed during field measurements.^45^

Finally, headgroup charge and oleic acid-oleate ratio depend on acidity. Aerosol particle acidity can vary with a typical pH range of ∼1–6, though this is difficult to measure directly and is normally inferred by applying theoretical models.^54−57^ Such a pH range would affect the degree to which weak acids, such as oleic acid (p*K*_a_ ∼ 5), are protonated. The surfactant headgroup charge affects the effective headgroup area which in turn can modify the curvature of a LLC phase.^29^ We simulated a change in headgroup charge by adding increasing amounts of sodium oleate to oleic acid, as would be found in less acidic conditions. Figure 3e demonstrates that increasing the headgroup charge (i.e., effective headgroup area) decreases the negative curvature of the headgroup-aqueous phase interface. This results in the following trend from low to high wt % sodium oleate: close-packed inverse micellar phase (*Fd*3*m* symmetry), inverse hexagonal phase (array of cylindrical micelles), and the lamellar phase.

Note that coexistent phases were also observed and were determined where more than one SAXS peak could be indexed unambiguously to a certain phase. Coexisting phases are an important feature of this study and have atmospheric implications (see Atmospheric Implications for a discussion).

### Changing the Nature of the Aqueous Phase

Soluble ionic species are components of atmospheric aerosols. Dissolved salts increase the ionic strength of a solution, which in turn increase the negative surfactant headgroup-aqueous phase interfacial curvature by shielding headgroup charge. As a result, molecular self-assembly can be influenced by the presence of dissolved inorganic species.^58^

Adding 1 wt % NaCl solution to the mixtures of varying sodium oleate content returned phases with higher negative curvature compared with the nonsaline aqueous phase (Figure 3a,b). Even at high sodium oleate content (80 wt %) the hexagonal phase prevailed. Increasing the salt concentration provides additional confirmation of this effect: an inverse hexagonal phase was observed in the salt-free mixture, whereas a more curved ordered inverse micellar phase (Fd3m symmetry) was observed at an aqueous phase salt concentration of 10 wt % NaCl (Figure 3c). An alternative explanation is that NaCl acts as a kosmotrope (water structure inducer), removing water from the headgroup-aqueous interface and stabilizing higher negative curvature interfaces.^58^ A change in aqueous phase salinity can therefore have a significant effect on the molecular arrangement of this proxy.

Atmospheric humidity plays an important role in determining aerosol properties such as phase state, affecting aging processes such as multiphase reactions and water uptake.^59^ We varied the water content of the base proxy mixture in order to simulate variations in aerosol water content. Filling the water cavity of an inverse topology phase is expected to induce the formation of phases with lower negative interfacial curvature, i.e., filling a spherical inverse micelle with increasing amounts of water will eventually induce cylindrical inverse hexagonal phase formation.^27,28^ The lamellar phase can be an exception to this trend: high and low water content lamellar phases can be produced without associated changes in curvature (see Figure 3d,e). Previous experiments on the oleic acid–sodium oleate proxy have focused on the anhydrous lamellar and crystalline lamellar phases.^20,21^

Figure 3a shows the progression from the more-curved close-packed inverse micellar phase to the less-curved inverse hexagonal phase with increasing water content. This is in line with the rationale outlined above. Furthermore, the hexagonal *d*-spacing (derived from the position of the SAXS peaks) increases with water content (see 30–50 wt % data points in Figure 3a). Note that the *d*-spacing stays roughly constant past 50 wt %, confirming that phases observed at water contents at or above this value are “excess water” phases, where the LLC coexists with external water that does not become incorporated into the cylindrical channels of the nanostructure; at this point, the cylinder radius corresponds to the lowest energy curvature. The close-packed inverse micellar phase is a viscous, translucent substance whereas the inverse hexagonal phase is opaque and diffusion of small molecules through its water channels is directionally dependent.^60^ A relatively small change in water content (from 10 to 30 wt %) resulted in a transition between these two molecular arrangements with quite different physical properties which would affect key aerosol processes such as water uptake and chemical reaction. A similar trend is observed when adding varying amounts of 1 wt % NaCl solution (Figure 3b). The maximum inverse hexagonal *d*-spacing is greater than that observed for the nonsaline aqueous phase, suggesting that the increase in ionic strength stabilizes larger water channels.

### Addition of Sugars

Fatty acids and sugars have been identified together as major constituents of particulate matter in both the urban and marine environments^3,61−63^ and the relative amounts of these molecules can vary on an hourly basis.^4^ This is a key motivation for the investigation of the effects sugar molecules have on the resulting fatty acid nanostructures in this proxy system.

**Figure 4:**
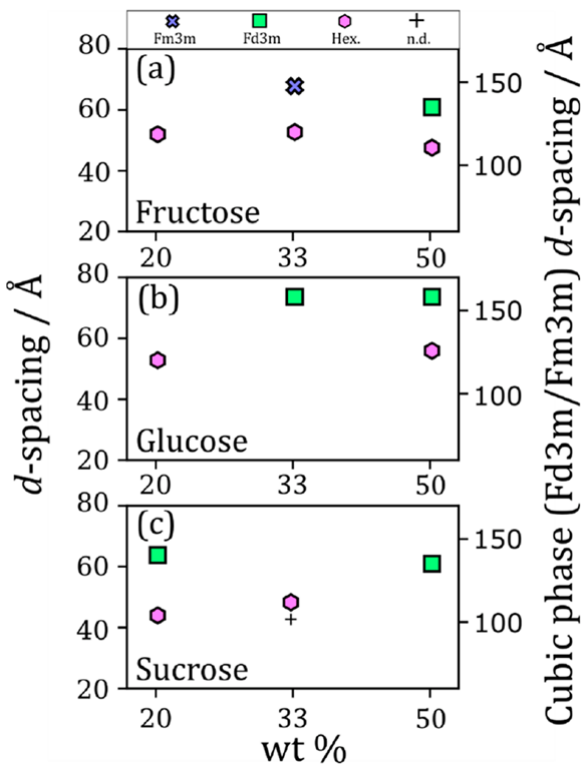
Plots of observed nanostructures and their calculated *d*-spacings vs wt % for each sugar added to the base mixture: oleic acid/sodium oleate (1:1) 50% wt in H_2_O. (a) wt % fructose; (b) wt % glucose; (c) wt % sucrose. The *d*-spacings for the cubic (*Fd*3*m* symmetry) nanostructure are placed on a different scale due to the large difference in values compared to the other nanostructures. Fru, fructose; Glu, glucose; Suc, sucrose. Phases are abbreviated: *Fm*3*m*-symmetry cubic close-packed inverse micelles (*Fm*3*m*); *Fd*3*m*-symmetry cubic close-packed inverse micelles (*Fd*3*m*); inverse hexagonal (Hex.); lamellar (Lam.); not determined due to overlapping peaks (n.d.).

As demonstrated in Figure 4, the amount and identity of the sugar clearly affects the fatty acid nanostructure. There is a trend common to all sugars studied: the negative curvature of the inverse phase increases with increasing sugar concentration, i.e. the nanostructure progresses from a cylindrical inverse hexagonal phase to a spherical ordered inverse micellar phase. We ascribe this to the sugars acting as kosmotropes, and removing water from the aqueous-surfactant headgroup interface and reducing the effective headgroup area.^58^

Fructose and glucose are closely related, having the same molecular mass. Despite this, the 33 wt % glucose and corresponding fructose mixtures returned ordered inverse micellar phases with differing symmetry (Figure 4a,b). This kind of variation in close-packed inverse micellar symmetry has been observed before in levitated particles of this proxy (without sugars), suggesting that these two symmetries are thermodynamically similar.^24^ These two symmetries must therefore be close together in the phase diagram for this system with and without sugars. Small differences in the exact composition or in the temperature of these mixtures could account for the observed variety in close-packing symmetry. The difference could also arise from the coexisting phases competing for water. However, it is difficult to deconvolute these hypotheses.

Sucrose, being a disaccharide, has been shown to be a stronger kosmotrope than glucose and fructose.^58^ This is demonstrated in Figure 4c, where at 20 wt % sucrose a close-packed inverse micellar phase is observed with a coexisting inverse hexagonal phase. This is not the case for the two monosaccharides (glucose and fructose), even though sucrose is about two times less concentrated in terms of molarity. Therefore, the identity of the sugar, in addition to the amount of sugar, has a marked effect on the nanostructures formed.

### Phase Changes in Levitated Droplets

LLC phase transformations can occur with changes in humidity. Levitating droplets of this proxy system and changing the surrounding humidity allows us to follow these changes in real time. The change in droplet water content also represents changes in solute concentrations and pH,^64,65^ which we indirectly varied in the bulk mixtures presented here. Therefore, the droplet-phase results presented here are more representative of what could occur in the atmosphere.

**Figure 5:**
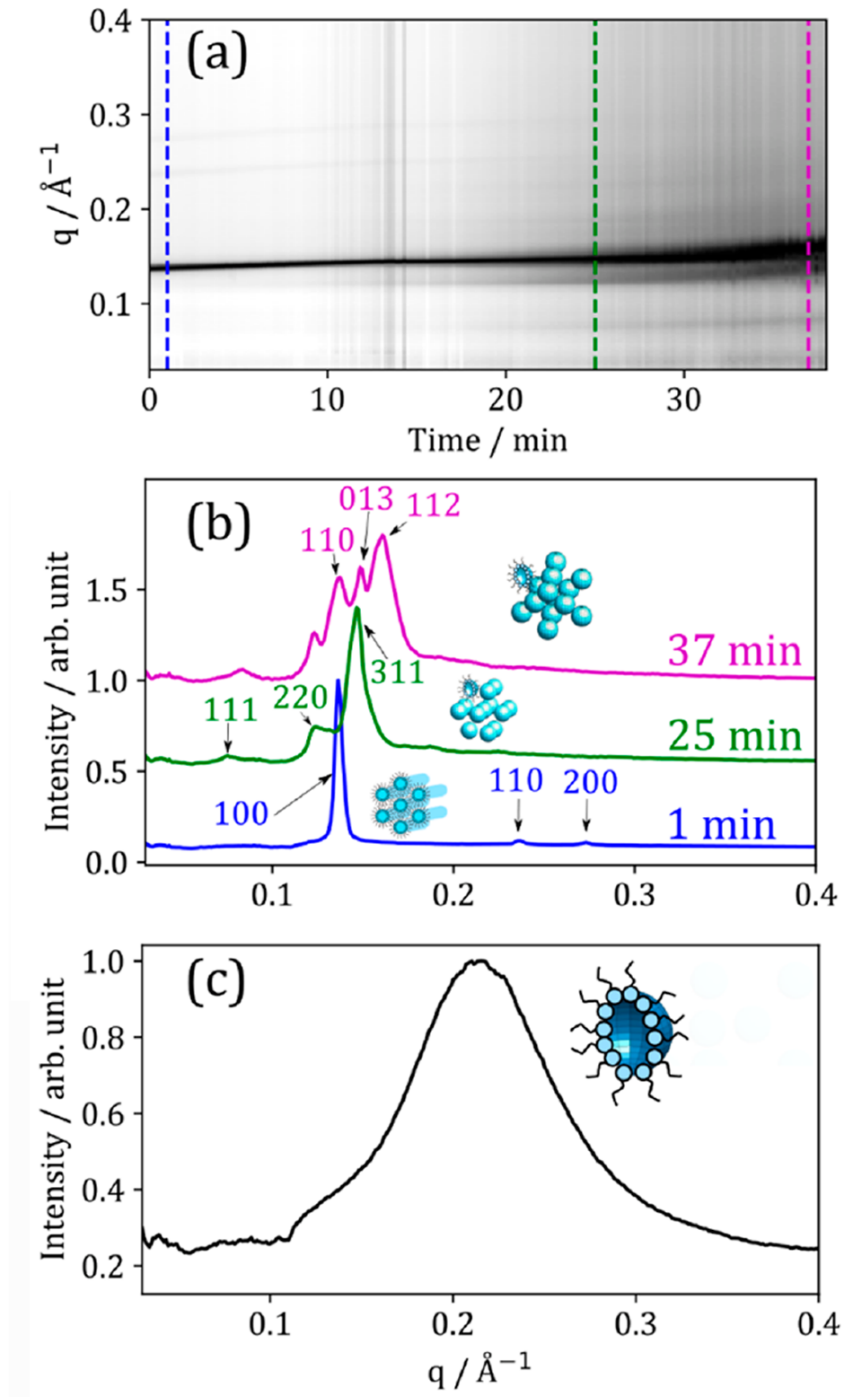
(a) One-dimensional SAXS pattern vs time during dehumidification from ∼86% RH to ∼12% RH. Colored dashed lines correspond to SAXS patterns in panel (b). (b) Selected 1D SAXS patterns from the same dehumidification experiment. The key Miller (*hkl*) indices for each phase (see Section S3, SI), along with a cartoon of each phase are labeled: inverse hexagonal (1 min); cubic close-packed inverse micelles (*Fd*3*m*); and hexagonal close-packed inverse micelles (*P*6_3_/*mmc*). (c) One-dimensional SAXS pattern from the center of the droplet after rehumidification from ∼12% RH to ∼83% RH.

Aqueous droplets containing oleic acid–sodium oleate and an excess of NaCl solution (1 wt %) were levitated at an elevated humidity (∼86% RH). The initial SAXS pattern revealed the hexagonal phase (blue trace in Figure 5a,b ). This is not the equilibrium phase at this RH. An experiment holding a droplet of this mixture at ∼86% RH for 20 min demonstrated a phase transition from the initial inverse hexagonal phase to a cubic close-packed inverse micellar phase (Figure S4, the SI).

Dehumidification to ∼12% RH returned close-packed inverse micelles (Figure 5a,b). Rehumidification of the sample environment to ∼83% RH returned a disordered inverse micellar phase consistent throughout the droplet (Figure 5c). The phase change therefore appears to be irreversible on this time scale (∼1 h).

The inverse hexagonal to close-packed inverse micellar transition is consistent with our bulk phase measurements; the close-packed inverse micellar phase occurs at a lower water content than the hexagonal phase (see Figure 3e). The NaCl concentration in the particle would also increase as water leaves the droplet, promoting the formation of close-packed inverse micelles. For bulk mixtures, the cubic close-packed inverse micellar phase (*Fd*3*m* symmetry) was commonly observed at higher NaCl concentrations before phase separation (see Figure 3d). This demonstrates that the trends observed in levitated droplets can be explained by observations in bulk mixtures.

### Five- and Six-Component Mixtures

**Figure 6:**
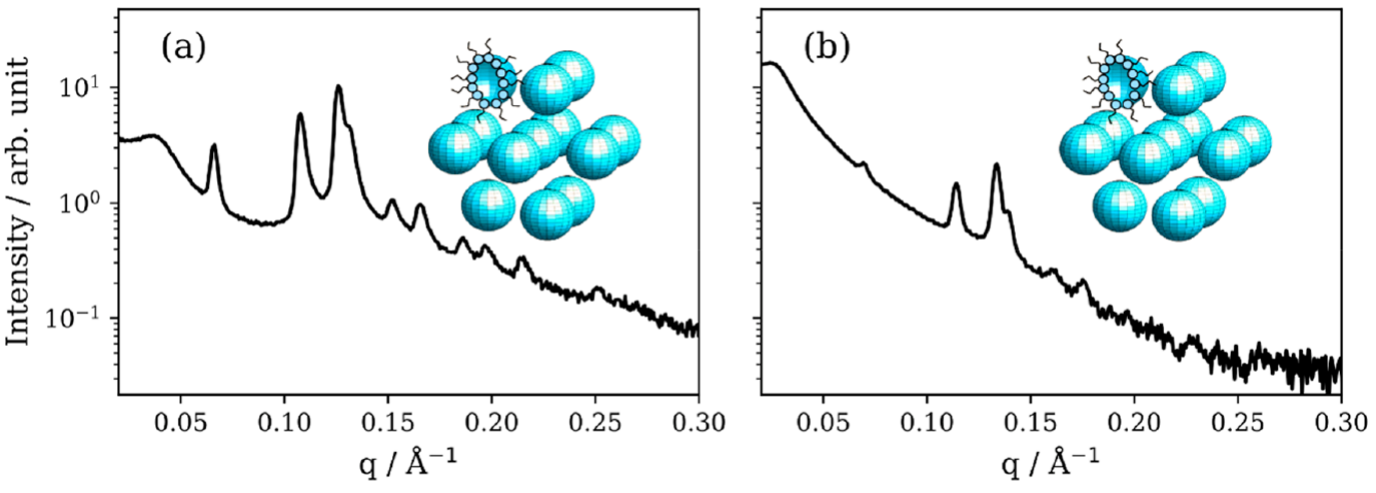
SAXS patterns measured on a laboratory instrument from five- and six-component mixtures. (a) Oleic acid–sodium oleate-fructose-glucose (1:1:1:1 wt) 50 wt % H_2_O, corresponding to a clear close-packed inverse micellar phase with Fd3m symmetry. (b) Oleic acid-sodium-oleate-fructose-glucose-sucrose (1:1:1:1:1 wt) 50 wt % H_2_O, corresponding to a close-packed inverse micellar phase with *Fd*3*m* symmetry. Both patterns exhibit a broad peak at low **q**, corresponding to coexisting disordered inverse micelles. Cartoons of each phase are presented.

We assessed the effect of adding more than one sugar to the base organic mixture. An inverse micellar cubic phase with *Fd*3*m* symmetry was observed for both mixtures (Figure 6a,b). The *d*-spacing was 166 Å for the five-component mixture, decreasing to 157 Å in the six-component mixture. Adding more components to the aqueous phase therefore decreases the size of the inverse micelles present in this structure. Details of how this was determined are in the SI.

The dominance of close-packed inverse micelles in these multicomponent mixtures was unexpected. A broad peak at low-**q** (∼0.02 Å^–1^) is most probably due to a coexistent disordered inverse micellar phase. This inverse micellar phase peak is more prominent for the six-component mixture. In both cases the close-packing of these inverse micelles imply an increase in viscosity, potentially by orders of magnitude^66^ that could be highly relevant in the atmospheric context (see Atmospheric Implications).

### Viscosity Changes

**Figure 7:**
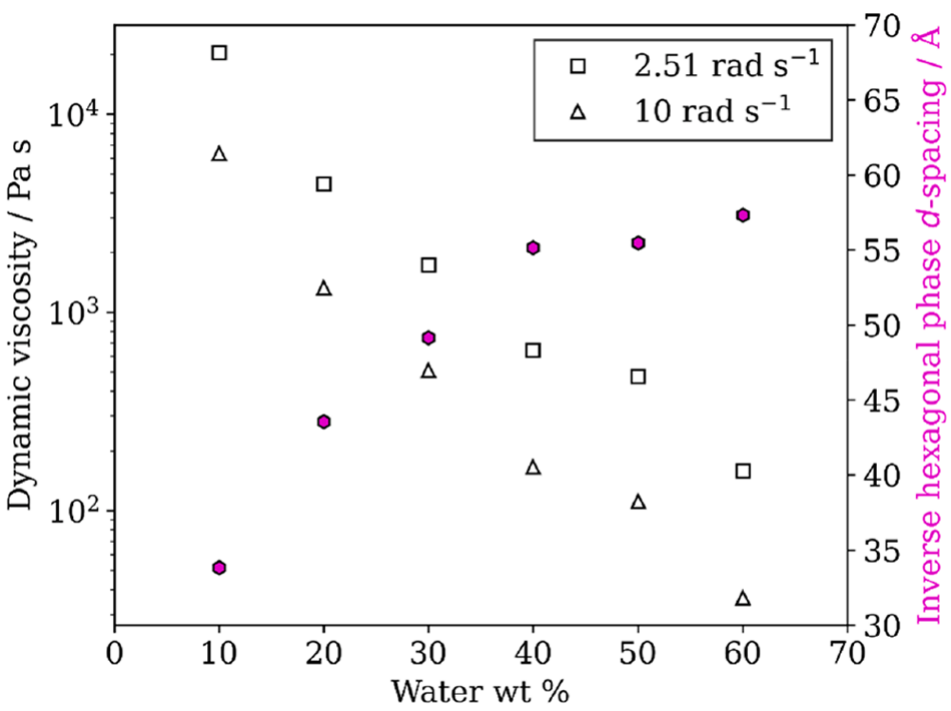
Dynamic viscosity of a 1:1 oleic acid–sodium oleate mixture vs water content (wt % water) at two different oscillatory frequencies. The corresponding *d*-spacing for the dominant inverse hexagonal phase observed in these mixtures is also presented.

The dynamic viscosity of the model 1:1 oleic acid–sodium oleate mixture decreased significantly with increasing water content, going from ∼10^4^ Pa s at 10 wt % water to ∼10^2^ Pa s at 60 wt % water (Figure 7). These values are firmly in the semisolid region^33,67^ and are consistent with what was predicted from kinetic modeling of self-organized oleic acid (∼10^2^ – 10^3^ Pa s).^22^ Although the mixtures used for rheology were dominated by the inverse hexagonal phase, they remained as unidentified coexisting phases. Therefore, we cannot ascribe these viscosities to a specific phase. This is, however, a more realistic representation of what might occur in the atmosphere.

The increased water content and subsequent increase in inverse hexagonal phase *d*-spacing clearly have a plasticizing effect on the mixture, allowing the organic phase to flow more freely.

### Atmospheric Implications

**Figure 8:**
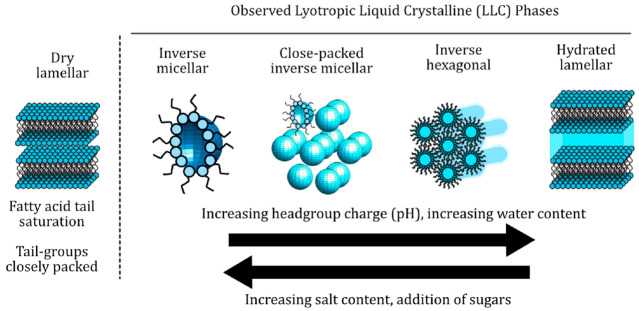
Nanostructures and the general trends observed in this study. Cartoons are not to scale.

Atmospheric aerosol composition is highly dynamic and will change with environment, season, time of day and emission source.^3,4,7,61,68^ In light of this, our results suggest that a variation in aerosol composition could have a marked impact on the resulting molecular arrangements of organic surfactants found in these aerosols; a major class of which are fatty acids, such as oleic acid studied here (Figure 8). These differences in nanostructure bring with them strongly differing physical properties. The trends observed here are consistent with what was predicted in a previous study of surfactant self-organization in this proxy.^24^

The viscosity of atmospheric aerosol particles affects water uptake and chemical reactivity, and these are two determiners of aerosol persistence and subsequent influence on the atmosphere.^32^ A range of aerosol phase states, from solid to liquid, is possible indoors and outdoors.^39−42^ Our work shows that differences in organic composition, water content, and salinity can produce changes in LLC phase and increasing the water content of the mixture causes a significant viscosity decrease. Viscosity can vary by orders of magnitude between nanostructures. For example, viscosity is roughly an order of magnitude higher for the inverse hexagonal phase compared with the close-packed inverse micellar (*Fd*3*m*) phase.^30,66^ Additionally, diffusion becomes anisotropic for the lamellar and inverse hexagonal phases; there is a significant difference between lateral and orthogonal diffusion in the lamellar phase.^31^ These variations in viscosity and diffusivity have implications for the uptake of trace gases (e.g., water vapor and atmospheric oxidants) into aerosol particles and can determine the time scale of bulk diffusion within them, impacting on aerosol persistence and atmospheric lifetime.^33−35,69,70^

Heterogeneity is a feature of many atmospheric aerosols. A range of imaging techniques have demonstrated that atmospheric aerosols are not always well-mixed.^71^ For example, organic coatings are present on marine aerosols,^13^ occasionally resulting in an inorganic core-organic shell morphology.^62^ Phase separation also happens in atmospheric aerosols^72,73^ and heterogeneities in particle viscosity have been observed to develop during the ozonolysis and humidification of aerosol proxies.^37,74^ During the present study, we observed an appreciable amount of nanostructural heterogeneity in the bulk mixtures. The most common coexisting phases were the inverse hexagonal and ordered inverse micellar phases. There are significant differences in physical characteristics between these two phases due to their different geometries. For example, the inverse hexagonal phase has directionally dependent water diffusion and is more viscous than the inverse micellar phase, observed by us qualitatively. We have shown that composition influences fatty acid nanostructure. If aerosol composition varies within a single aerosol particle, there could be a similar spatial heterogeneity in fatty acid nanostructure, impacting aerosol processes and subsequent effects on urban air pollution and climate through variations in the time scales of aerosol trace gas uptake due to the implied viscosity differences.^33^

Aerosol optical properties can vary with environment and over time^5^ with aerosol light scattering and absorption contributing to radiative forcing.^2^ We qualitatively found visual differences between the optically isotropic phases with cubic symmetry (close-packed inverse micellar) and optically anisotropic phases (lamellar and inverse hexagonal) (also compare Hyde, 2001);^75^ the former was translucent and the latter was opaque. This observation suggests that aerosol light scattering could be affected by the optical characteristics of the fatty acid nanostructures, resulting in an additional indirect effect on the climate.

## Conclusions

We have explored the molecular arrangements accessible to the oleic acid–sodium oleate fatty acid aerosol proxy in levitated droplets and bulk mixtures. This was achieved by addition of other common atmospheric emissions (sugars and saturated fatty acids) and by adjusting the amount of water and salinity of the aqueous phase. Notably, the composition of the proxy heavily influenced the resulting nanostructure. Over a small range of aqueous phase salinity, a phase transition was observed between two nanostructures known to have different physical characteristics and phase changes were also observed in levitated droplets. The viscosity was observed to decrease by orders of magnitude with increasing water content.

Bridging the gap between simple aerosol proxy systems and real atmospheric measurements continues to be a challenge (compare, e.g., Shepherd et al., 2022; Woden et al., 2021).^25,76^ However, this study is a step toward linking the laboratory with the real world when considering the relatively novel proposition of LLC phase formation in the atmosphere.

The potential importance of fatty acid self-organization in the atmosphere is added to by the demonstration of the range of (often coexisting) nanostructures observed in this study both in bulk mixtures and levitated droplets, and even in the more complex mixtures with up to six components we tested here. We now have better knowledge of the phase space accessible to these molecules, providing a basis for future, more quantitative investigations into the physical characteristics of the nanostructures discussed in this study and likely encountered in the atmosphere.

## Supporting Information Available

Phase-composition table, a WAXS pattern of the oleic acid-stearic acid mixture, indexing calculations for the observed cubic phases, and additional levitation-SAXS data (PDF)
