# Supplementary material for: Exploring the Nanostructures Accessible to an Organic Surfactant Atmospheric Aerosol Proxy
Source: J Phys Chem A. 2022 Sep 28;126(40):7331–41. doi: 10.1021/acs.jpca.2c04611 (PMC9574911; doi:10.1021/acs.jpca.2c04611)
Supplement: Supplementary file 1 — jp2c04611_si_001.pdf [file jp2c04611_si_001.pdf]

Supporting information for “Exploring the Nanostructures Accessible to an Organic Surfactant Atmospheric Aerosol Proxy”

Adam Milsom[1], Adam M. Squires[2], Isabel Quant[2], Nicholas J. Terrill[3], Steven Huband[4], Ben Woden[5], Edna R. Cabrera-Martinez[5] and Christian Pfrang[1,6*].

[1]School of Geography, Earth and Environmental Sciences, University of Birmingham, Edgbaston, B15 2TT, Birmingham, UK.
[2]Department of Chemistry, University of Bath, South Building, Soldier Down Ln, Claverton Down, BA2 7AX, Bath, UK.
[3]Diamond Light Source, Diamond House, Harwell Science and Innovation Campus, OX11 0DE, Didcot, UK.
[4]Department of Physics, University of Warwick, Coventry, CV4 7AL, UK.
[5]Department of Chemistry, University of Reading, Whiteknights, Reading, RG6 6AD, UK.
[6]Department of Meteorology, University of Reading, Whiteknights, Earley Gate, RG6 6BB, Reading, UK
*E-mail: c.pfrang@bham.ac.uk

S1. Wide-angle X-ray scattering (WAXS) pattern of an oleic acid-stearic acid mixture

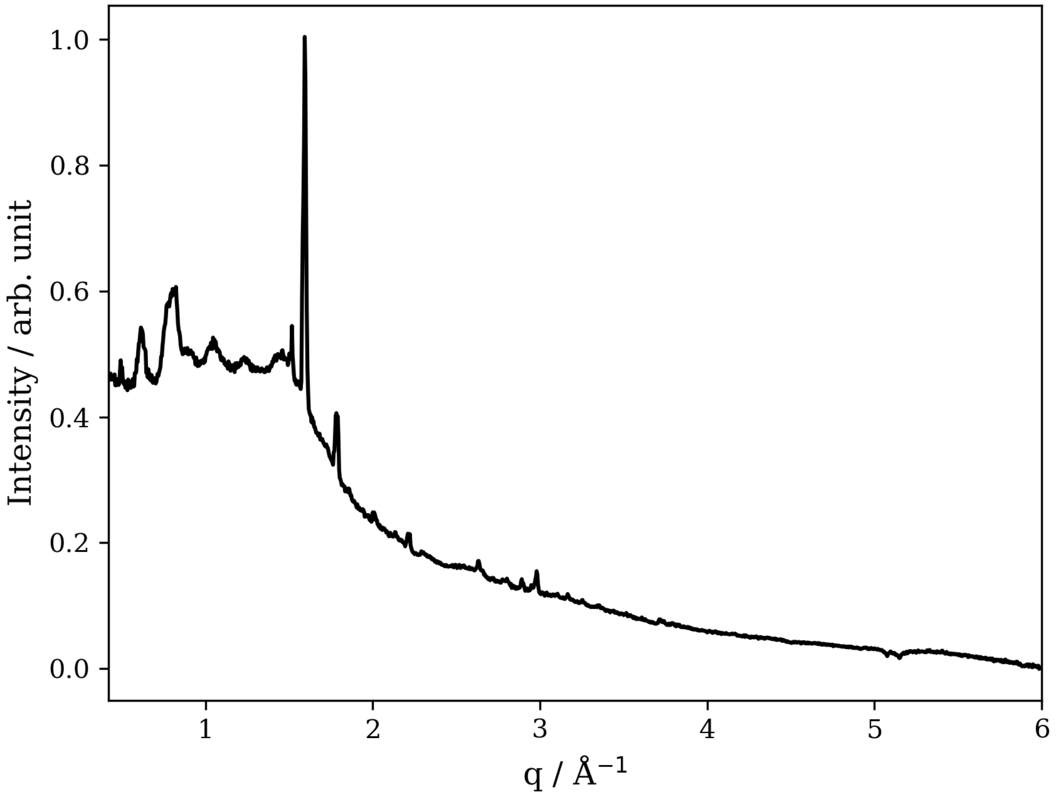

Figure S1. WAXS pattern of a 1:1 wt oleic acid-stearic acid mixture, background subtracted. The sharp peaks observed here correspond to the short-distance correlations between adjacent alkyl chains in the crystalline lamellar phase formed by this mixture.

S2. Phase-composition data

**Experiment series (figure in main text)**	**wt % of organic mixture**			**aqueous phase wt %**		
Stearic acid + oleic acid (Fig. 3(d))	*OA*	*SO*	*StA*	*H_2O*	*Phase(s) observed*	*d-spacing(s)*
	20	0	80	0	Lam.	41
	50	0	50	0	Lam. | Lam.	41 | 40
	80	0	20	0	Lam. | Lam.	41 | 39
Sodium oleate in H_2O (Fig. 3(e))	*OA*	*SO*		*H_2O*	*Phase(s) observed*	*d-spacing(s)*
	80	20		50	Fd3m	135
	66	33		50	Fd3m | Hex.	147 | 48
	33	66		50	Hex.	54
	29	71		50	Lam.	92
	20	80		50	Lam. | Mic. (phase separation)	45
Sodium oleate in 1% NaCl (Fig. 3(f))	*OA*	*SO*		*NaCl (1%)*	*Phase(s) observed*	*d-spacing(s)*
	66	33		50	Fd3m	127
	33	66		50	Hex. | n.d.	55
	20	80		50	Hex.	52
[NaCl] (Fig. 3(c))	*OA*	*SO*		*NaCl (% concentration)*	*Phase(s) observed*	*d-spacing(s)*
	50	50		0	Hex.	52
	50	50		5	Fd3m	110
	50	50		10	Fd3m	138
	50	50		20	Fd3m | Lam. (phase separation)	101 | 45
	50	50		60 (saturated)	Lam. | Mic. (phase separation)	45 | 33
wt % H_2O (Fig. 3(a))	*OA*	*SO*		*H_2O*	*Phase(s) observed*	*d-spacing(s)*
	50	50		10	Fd3m | n.d.	114
	50	50		30	Hex.	41
	50	50		50	Hex.	54
	50	50		70	Hex.	55
	50	50		90	Fd3m | n.d.	160
wt % NaCl (1%) (Fig. 3(b))	*OA*	*SO*		*NaCl (1%)*	*Phase(s) observed*	*d-spacing(s)*
	50	50		10	Mic. | Lam.	29 | 41
	50	50		30	Hex.	40
	50	50		70	Hex.	54

	50	50		90	Hex.	42.7
wt % fructose (Fig. 4(a))	*OA*	*SO*	*Fructose*	*H_2O*	*Phase(s) observed*	*d-spacing(s)*
	50	50	20	50	Hex.	52
	50	50	33	50	Hex. | Fm3m	49 | 68
	50	50	50	50	Hex. | Fd3m	48 | 135
wt % glucose (Fig. 4(b))	*OA*	*SO*	*Glucose*	*H_2O*	*Phase(s) observed*	*d-spacing(s)*
	50	50	20	50	Hex.	53
	50	50	33	50	Fd3m	158
	50	50	50	50	Hex. | Fd3m	56 | 158
wt % sucrose (Fig. 4(c))	*OA*	*SO*	*Sucrose*	*H_2O*	*Phase(s) observed*	*d-spacing(s)*
	50	50	20	50	Hex. | Fd3m	44 | 140
	50	50	33	50	Hex. | n.d.	52
	50	50	50	50	Fd3m	135

Table S1. Composition-phase-*d*-spacing data supporting the figures presented in the main text. The observed phases are abbreviated as follows: Inverse micellar (Mic.); close-packed inverse micellar with Fd3m symmetry (Fd3m); close-packed inverse micellar with Fm3m symmetry (Fm3m); inverse hexagonal (Hex.); Lamellar bilayers (Lam.); not determined due to hidden/overlapping peaks in the SAXS pattern (n.d.).

S3. Indexing the Fd3m phase formed by the 5- and 6- component mixtures

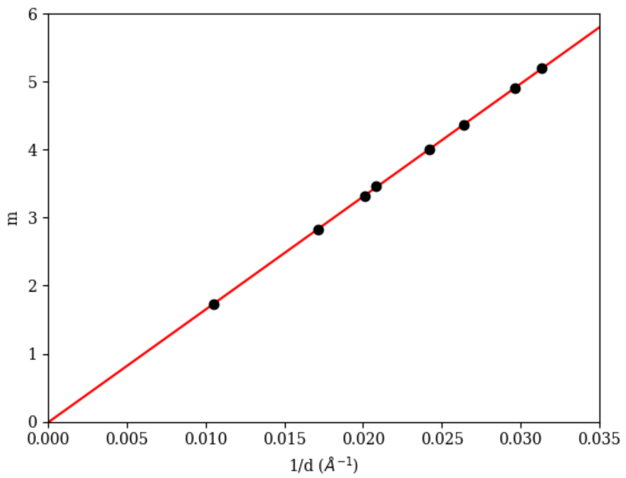

Figure S2. A plot of *m* vs 1/*d* for each observed reflection for the oleic acid-sodium oleate-glucose-fructose (1:1:1:1 wt) 50 wt % H_2O mixture, showing a linear plot with an origin at 0. The Fd3m symmetry.

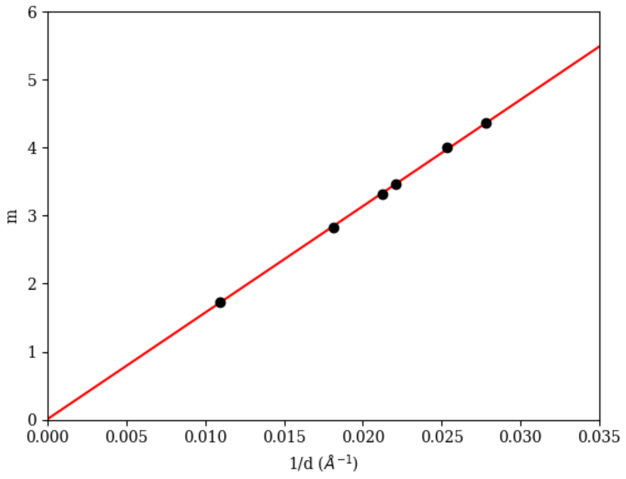

Figure S3. A plot of *m* vs 1/*d* for each observed reflection for the oleic acid-sodium oleate-glucose-fructose-sucrose (1:1:1:1:1 wt) 50 wt % H_2O mixture, showing a linear plot with an origin at 0. The Fd3m symmetry.

A relationship between the Miller indices (*hkl*) of each scattering plane and 1/*d* for each observed reflection is defined:

$$m = \sqrt{(h^2 + k^2 + l^2)}$$

The parameter, *m*, is unique to Fd3m symmetry group. A linear relationship between *m* and experimentally observed peak positions 1/*d* with an intercept at 0 would mean that the phase assignment is correct [1].

Both the 5- and 6- component mixtures return linear plots with intercepts at 0, confirming the cubic close-packed inverse micellar phase for these mixtures. The lattice parameter for each is 166 and 157 Å for the 5- and 6-component mixtures, respectively.

S4. SAXS experiment on a levitated droplet held at ~ 86 % RH

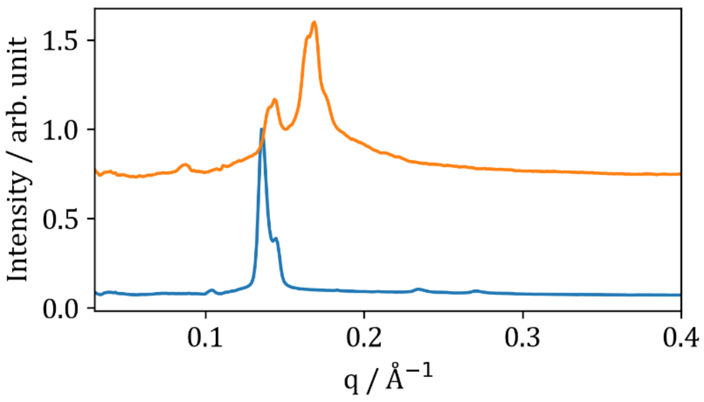

Figure S4. 1-D SAXS patterns of a levitated droplet being held at ~ 86 % RH. Initial inverse hexagonal phase observed with a co-existing phase (bottom). Cubic close-packed inverse micellar phase (Fd3m) after being held at the same RH for 20 min (top).
